# Velocity-dependent shear band formation in blanking processes

**DOI:** 10.1038/s41598-026-51972-3

**Published:** 2026-05-06

**Authors:** Lisa Winter, Sven Winter, Katja Martinitz, Olaf Schrage, Luisa Schottstedt, Marcus Böhme, Mario Scholze, Marlon Hahn, Rico Drehmann, Verena Psyk, Martin F.-X. Wagner, Wolfram Volk, Yannis P. Korkolis, Thomas Lampke

**Affiliations:** 1 Institute of Materials Science and Engineering, Materials and Surface Engineering Group, Chemnitz University of Technology, 09107 Chemnitz, Germany; 2 Fraunhofer Institute of Machine Tools and Forming Technology IWU, Reichenhainer Straße 88, 09126 Chemnitz, Germany; 3 Chair of Metal Forming and Casting, Technical University of Munich, Walther- Meissner-Strasse 4, 85748 Garching, Germany; 4 Institute of Forming Technology and Lightweight Components, Dortmund University, Baroper Str. 303, 44227 Dortmund, Germany; 5 Institute of Materials Science and Engineering, Chemnitz University of Technology, 09107 Chemnitz, Germany

**Keywords:** High-speed blanking, Blanking speed, Low-alloyed steel, Adiabatic shear band (ASB), Drive concept, Shear strain rate, Engineering, Materials science, Physics

## Abstract

High-speed blanking processes are of major interest for manufacturing sheet components from high-strength materials, as not only high-quality surfaces but further excellent properties are enabled. Reason for this are adiabatic shear bands (ASB) formed in the shear zone during the process. However, up to now it is not fully understood how ASBs evolve depending on the process conditions. For the first time, surface geometry and ASB formation are investigated for an identical macroscopic stress state using different blanking speeds. A press-hardened 22MnB5 steel is blanked with punch velocities ranging from 0.12 to 17 m/s using different machine drive concepts (mechanical, hydraulic and electromagnetic), resulting in nominal shear strain rates of up to nearly 100,000,000 s⁻¹. The velocity significantly influences ASB length and width as well as the microstructure and hardness of the blanked surfaces. For 17 m/s blanking speed, an ASB covering the full-length of the blanked surface, i.e. the complete sheet thickness, is realized. Further, for high velocities, ASBs not only dominate the appearance of the blanked surface but also lead to significantly lower roughness, when compared to conventionally blanked surfaces. This study lays the foundation for future work on tailoring functional surfaces using high-speed blanking.

## Introduction

 High-speed blanking (HSB), also known as high-speed impact cutting (HSIC) or adiabatic blanking, not only allows for the processing of highly ductile materials as well as high-strength steels^1^, but further leads to excellent surface qualities of the blanked materials^2^. Mostly, blanking with speeds above 0.8 m/s is referred to as HSB. To enable these high blanking speeds, various drive concepts can be used. Mechanical presses using special tools can already achieve the lower speed limit of approximately 1 m/s^3^. To date, high-speed hydraulics with maximum speeds of approximately 10 m/s have been used most frequently^4^. Currently, compact electromagnetic drives are being tested because they can achieve significantly higher speeds of more than 15 m/s^5^. Especially for materials such as high-strength steels, the high-speed blanked surface shows adiabatic shear bands (ASBs) as characteristic feature. Formation and evolution of these ASBs have been subjects of various research activities^6–8^. In these mesoscopic, band-like structures, nanocrystalline grains are present and the ASB exhibits a higher resistance against etching, when compared to the surrounding microstructure of the material^9^. For this reason, ASBs were formerly referred to as “white etching bands”^10^. Adiabatic shear bands are a result of a localized deformation, causing dynamic recrystallization (DRX)^11–13^, which is driven by high hydrostatic pressure in the shear zone, shear stress, and temperature resulting from the high blanking speed^2^. Due to their microstructure, ASBs have unique properties that can lead to improved mechanical strength, geometrical accuracy, and tribological as well as corrosion resistance of the blanked edge of a component, allowing them to be used as functional surfaces in industrial applications without post-processing^14^. However, to directly control the blanking edge properties and fulfill the requirements of the specific application, understanding of the complex interactions between the process parameters, e.g. blanking speed, clearance, resulting stress state and triaxiality, and the resulting microstructure, e.g. shear band formation and evolution, is absolutely necessary. In the present study, for a press-hardened steel the effect of the blanking speed in an extremely wide speed range on the formation of ASBs and the microstructure of the blanked edge, which is influenced by the induced shear deformation, is investigated for the first time to the best of our knowledge. It is shown that, for an identical macroscopic stress state defined by the sheet thickness, the clearance of the tool and the geometry of the blanked part, the geometry of the blanked surface as well as the presence and appearance of ASBs is significantly influenced by the blanking speed.

## Methods

**Fig. 1:**
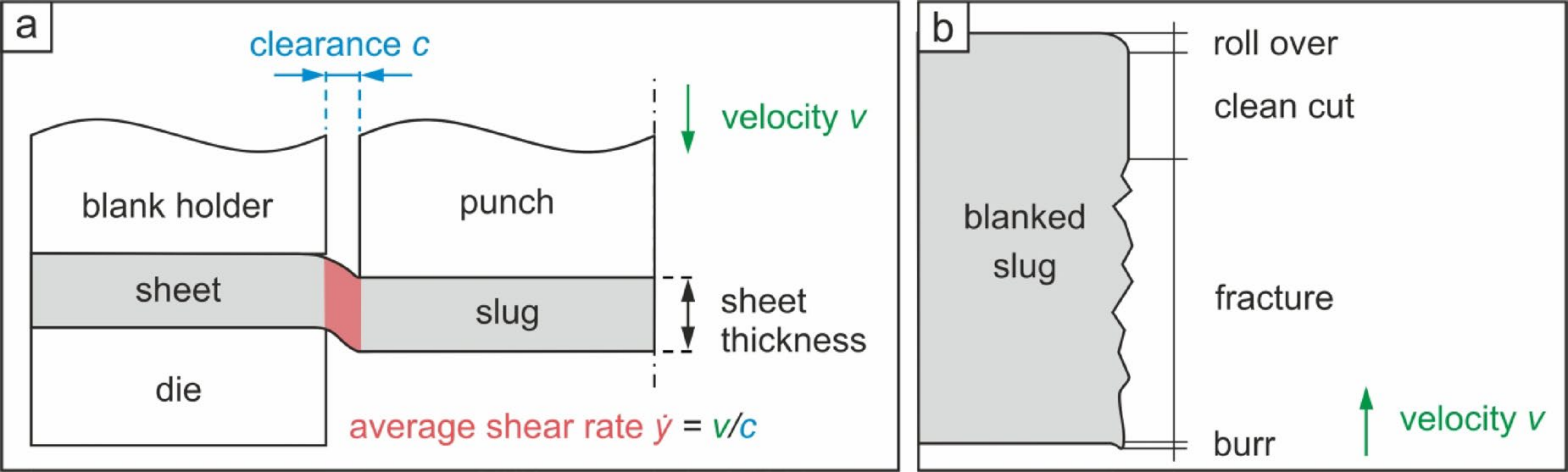
Schematic representations of the (**a**) blanking process (shear zone with average shear rate calculated from clearance and velocity is marked in red) and (**b**) cross section of blanked surface according to VDI 2906^17^ (the slug has to be flipped, which is displayed by the velocity arrow).

In this study, a 22MnB5 steel (C = 0.186 wt.-%; Mn = 1.039 wt.-%; Si = 0.223 wt.-%; *P* = 0.007 wt.-%; B = 0.001 wt.-%; Fe = balance, conventionally used in automotive engineering^15^, with a sheet thickness of 2 mm and a hardness of approx. 600 HV was investigated. The sheet material, austenitized at 930 °C for 5 min and subsequently press-hardened in a water-cooled tool, was supplied by Salzgitter Mannesmann Forschung GmbH). Blanking was carried out at participating research institutions using variable drive concepts. The combined use of different drive concepts is a necessity to enable the investigation in a wide speed range, as each specific drive concept is somewhat limited in the realizable blanking speed. Blanking speeds of 0.12 and 0.7 m/s were realized using a conventional BRUDERER BSTA 1600 high-performance stamping press with a mechanical drive^3^. 8 m/s blanking speed was realized with an ADIAClip from MPM France with a hydraulic drive^16^. The highest velocity of 17 m/s, was achieved using an electromagnetically accelerated tool, as described in^5^. The principal setup of the experiment was identical for all tests and all speeds to enable a methodologically sound comparison between the different drive concepts, as shown schematically in Fig. 1a. In addition to the blanking speed, the most important parameter is the clearance *c*, which is defined as the distance between the die and the punch. The clearance determines the stress state, as well as the nominal shear strain rate in the shear zone (Fig. 1a, red area). The relative clearance, referred to the sheet thickness, was 5% in all tests, which corresponds to a clearance of 100 μm. The resulting nominal shear rate assigned to the blanking speeds varies from 1,200 to 170,000 s⁻¹. Figure 1b shows a schematic representation of the resulting blanked surfaces according to VDI 2906^17^, which is commonly used to evaluate surfaces produced by conventional or fine blanking processes. In the present study, only the slugs (rotationally symmetrical coins with a diameter of 20 mm) were considered and their metallographically polished cross sections were used to evaluate the blanked surfaces. As the representation according to VDI 2906 defines the alignment of clean cut to fracture zone from top to bottom, the slug has to be flipped upside down for the micrographs, when compared to the blanking direction (see orientation of velocity arrow in Fig. 1b). The metallographically polished cross sections of the blanked slugs were analyzed by optical microscopy. Further, the roughness of the blanked surfaces of the slugs was determined using a 3D laser scanning microscope Keyence VK X200 and an area of 2 × 2 mm^2^ (section of circumference x slug thickness) was measured. Evaluation of roughness values was done in accordance to ISO 21,920^18^ in the regions 400 μm near to the upper and lower edge of the sheet, which correspond to the clean cut and fracture area represented by VDI 2906.

In addition, for the lowest and the highest blanking speeds, the shear zone at the blanked surface was analyzed further. Electron backscatter diffraction (EBSD) was performed using a field emission scanning electron microscope ZEISS GeminiSEM 460 equipped with an Oxford Instruments Symmetry S2 EBSD Camera. The cross sections for EBSD analysis were ground (to 2500 grit on SiC-paper) and polished. A final polishing was executed with a vibratory polisher using colloidal silica suspension. The EBSD measurement was performed at 20 kV with a step size of 0.1 μm for the overview scans and at 15 kV with a step size of 0.06 μm for the detail scans. Data evaluation was done using the Oxford AZtec Crystal software (version 3.3) after a slight clean-up procedure (wild spikes removal). The detail scans were analyzed with regard to grain orientation spread (GOS) and grain area (GA) using the mean value from ten line measurements, each 25 pixels wide.

To determine the hardness depending on the microstructure for these two conditions micro hardness HV0.001 was measured using a Fischersope HM2000 XYm. Four line measurements from the embedding resin through the shear zone towards the undeformed steel substrate were done with a loading force of 9,8 mN and a distance of 10 μm between individual indents.

## Results and discussion

**Fig. 2:**
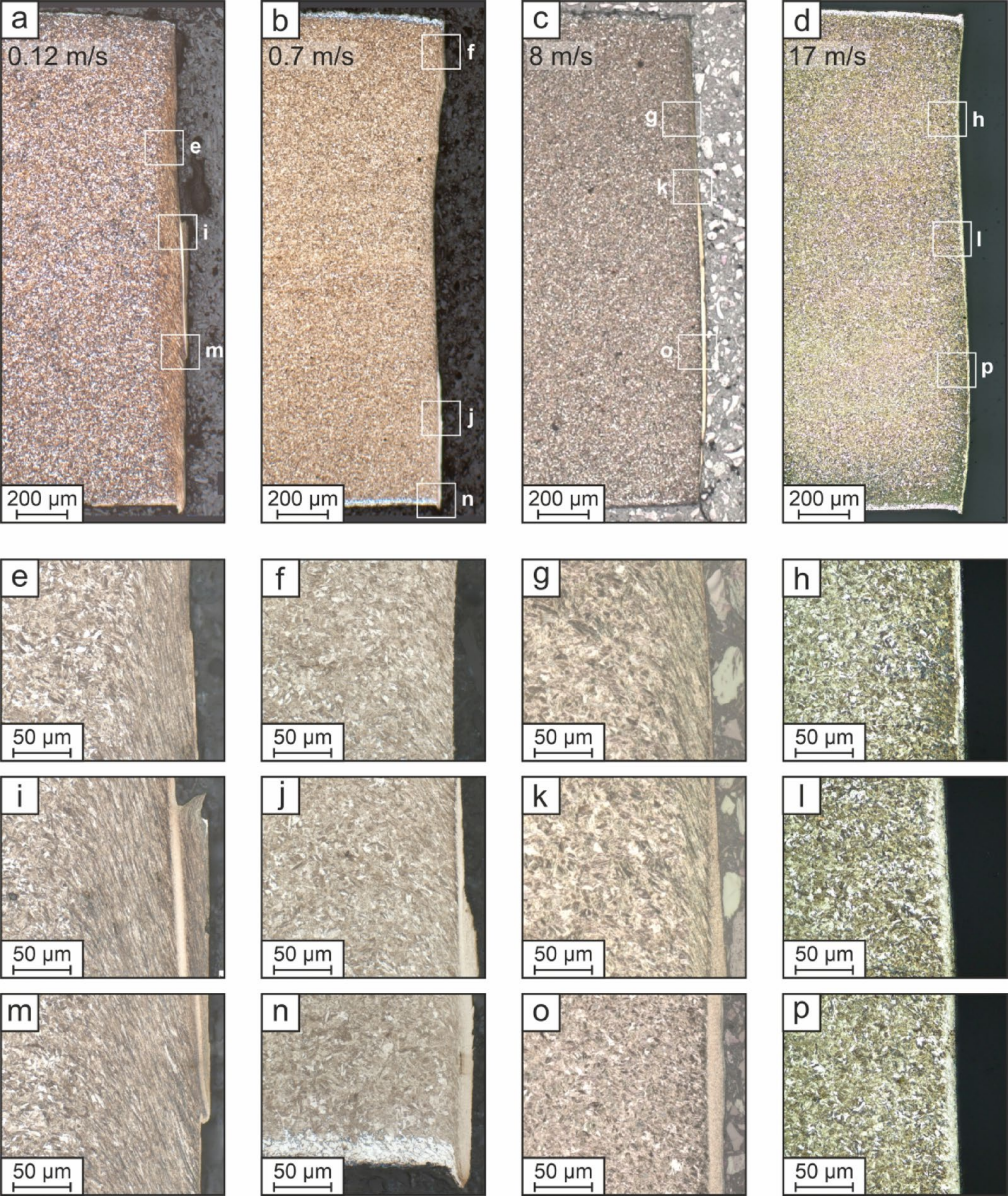
Optical micrographs of the surfaces blanked at (**a**) 0.12, (**b**) 0.7, (**c**) 8 and (**d**) 17 m/s speed. Higher magnified images for a blanking speed of (**e**, **i**, **m**) 0.12, (**f**, **j**, **n**) 0.7, (**g**, **k**, **o**) 8 and (**h**, **l**, **p**) 17 m/s. An increase in blanking speed leads to a pronounced S-shaped profile of the blanked surface with a much smoother appearance.

The optical micrographs of the blanked surfaces resulting from the four investigated blanking speeds are compared in Fig. 2, with the complete blanked surfaces shown in Fig. 2a–d and details focusing on the deformation zone and the ASB width displayed in Fig. 2e–p, respectively (marked with white rectangles in Fig. 2a–d). Obviously, the blanking speed has a significant influence on the geometry of the surface and, further, the formation of ASBs.

The white edges, which can be detected near to the top and bottom surface of all sheets, indicates decarburization resulting from the press-hardening process of the steel material. Table 1 lists the roughness of the blanked surfaces depending on the blanking speed.

Figure 2a shows the results for the slowest punch velocity of 0.12 m/s, which corresponds to a conventional blanking process. Despite the high hardness and the fully martensitic microstructure of the press-hardened steel sheet, a pronounced deformation zone is obvious for the whole blanked edge. Further, even for the lowest investigated engineering shear strain rate of 1,200 s⁻¹ at this blanking speed, an ASB occurs in the center of the blanked surface. In literature studies, a shear strain rate of 1,000 s⁻¹s is mostly stated as minimum depending on the specimen geometry and the stress state for the formation of ASBs^19^. Typically, failure is expected to occur in the thermally softened ASB^20,21^. However, this is not the case for the surface blanked at 0.12 m/s, as the higher magnified images in Fig. 2e, i, m show failure occurring next to the shear band near the deformation zone. Presumable, this failure location would lead to a blanked sheet as counterpart, where only deformation zone and uninfluenced martensitic microstructure is observable. Further, the ASB has a jagged appearance with a maximum width of 7.6 μm and a length of 575 μm. Considering the roughness of the surface blanked at 0.12 m/s, it is the highest for all investigated blanking speeds (see Table 1).

In Fig. 2b, the blanked surface resulting from a speed of 0.7 m/s, which corresponds to a shear rate of 7,000 s⁻¹, is shown. The geometry of the blanked surface is completely different, when compared to the one resulting from the lower blanking speed and the characteristic features are not any longer describable using VDI 2906. For 0.7 m/s the profile of the blanked surface is slightly concave, and the deformation zone is smaller (see Fig. 2f, j, n). As for the lower blanking speed, the width of the deformation zone is largest more near to the center of the slug. An ASB with a maximum width of 8.3 μm and a length of 531 μm is detectable nearer to the lower sheet edge in the blanked surface. Further, the ASB exhibits jagged areas and a very inhomogeneous thickness (see Fig. 2j, n). Comparing to the roughness of the surface blanked at punch velocities of 0.12 m/s, the roughness of the surface blanked at 0.7 m/s is almost halved (see Table 1).

Figure 2c shows the surface after blanking at a speed of 8 m/s. The corresponding surface appearance with an S-shaped profile is commonly reported in literature as a result of high-speed blanking^2,16,22^. The pronounced S-shape is attributed to the formation of longer ASBs. At a nominal shear rate of 80,000 s⁻¹ corresponding to a blanking speed of 8 m/s, the ASB has a maximum width of 13.3 μm and a length of 1,285 μm, which corresponds to more than the half of the sheet thickness (see Fig. 2o). Failure seems to occur very close to or within the ASB (see Fig. 2g, k). Blanking with this speed results in a roughness of the blanked surface which is even lower than that of the surface blanked at 0.7 m/s and additionally features smaller deviations of the roughness values (see Table 1). The maximum width of the deformation zone is comparable for 0.7 and 8 m/s. In areas without ASB, the deformation zone is much more pronounced (see Fig. 2f, g).

For the highest investigated blanking speed of 17 m/s (corresponding to a nominal shear rate of 170,000 s⁻¹), the blanked surface exhibits an ASB covering the full length of the sheet thickness (see Fig. 2d). Surprisingly, the ASB width seems to be much more homogenous and the maximum width is significantly smaller with 8.1 μm, when compared to the ASB resulting from a blanking speed of 8 m/s. Further, no deformation zone is detectable (see Fig. 2h, l, p). Presumably, failure occurred in the ASB as no jagged crack path is observable. However, this would only be detectable by analyzing the blanked sheet as well. In case of failure directly in the ASB, length and width of the ASB would be equal for the slug and the blanked sheet. The S-shaped profile of the blanked surface dominates the geometric properties and is much more pronounced, when compared to the surface blanked at 8 m/s. Regarding the roughness, it seems that the trend of lower roughness with an increasing blanking speed saturates as the roughness values are similar to the surface roughness resulting from 8 m/s blanking speed. Nonetheless, this demonstrates the high quality of blanked surfaces with a pronounced proportion of ASB, which are formed by high-speed blanking.

**Table 1:** Roughness of the blanked surfaces resulting from the different investigated blanking speeds determined by laser scanning microscopy.

Blanking speed v in m/s		0.12	0.7	8	17
Roughness Ra in µm	400 μm from upper edge	6.10 ± 0.62	4.13 ± 1.63	3.67 ± 0.06	4.43 ± 0.25
	400 μm from lower edge	9.86 ± 0.29	3.75 ± 0.33	3.82 ± 0.11	3.69 ± 0.37

**Fig. 3:**
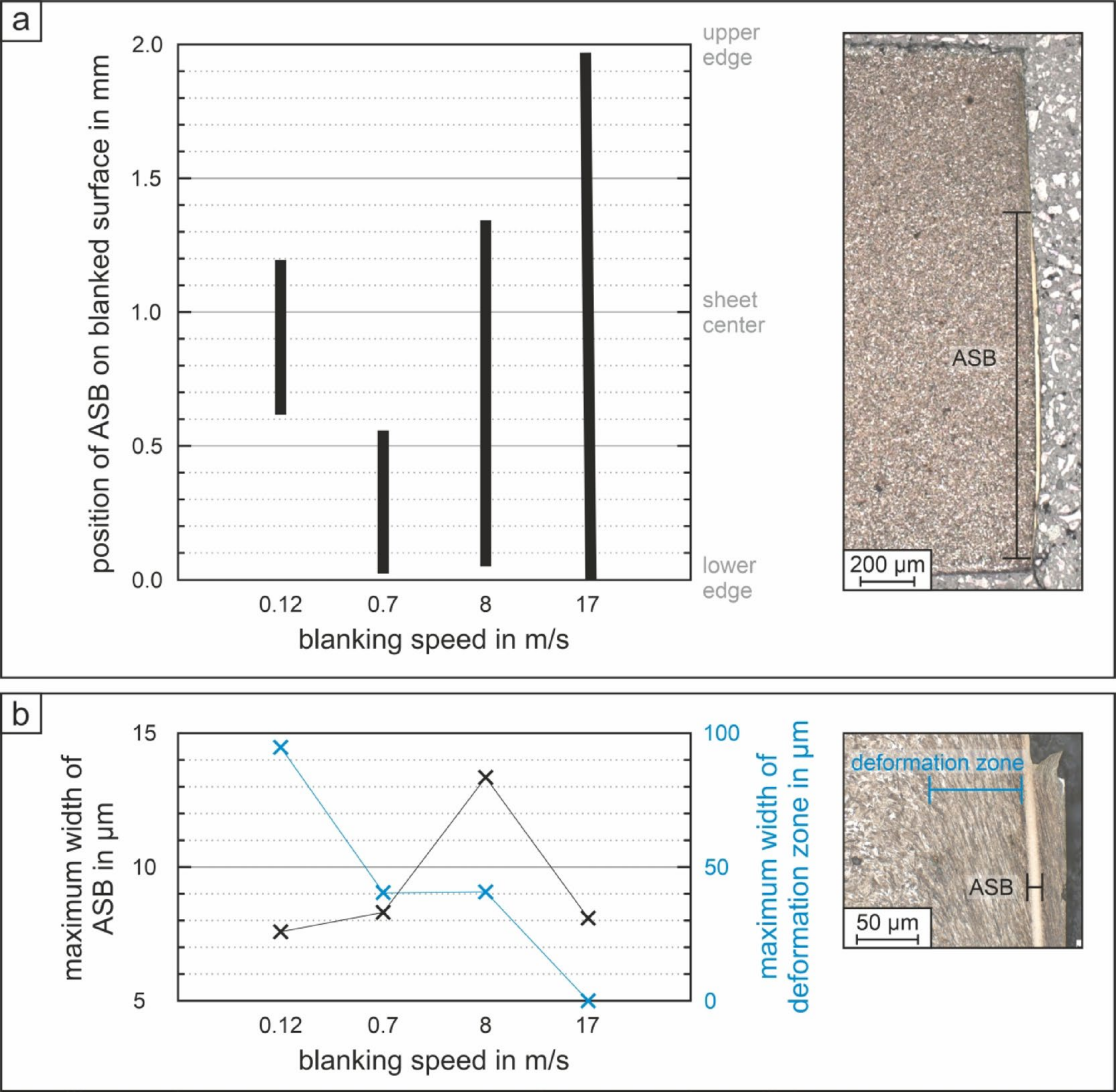
Schematic evaluation based on the optical micrographs of (**a**) ASB length over blanked surface, respectively sheet thickness, and (**b**) maximum width of the deformation zone and the ASB depending on the investigated blanking speed. With an increase in blanking speed, the ASB length increases and the width of the deformation zone decreases. For the highest blanking speed of 17 m/s, the ASB is formed over the entire blanked surface, without a deformation zone.

Figure 3 gives a schematic representation of the summarized results in which Fig. 3a illustrates how the position and length of the ASB on the blanked surface change depending on the blanking velocity. Clearly, lower velocities result in significantly shorter ASBs. For blanking velocities of 8 m/s and above, the ASB dominates the blanked surface, which shows a pronounced S-shaped profile. In Fig. 3b, the maximum width of the deformation zone next to the ASB and the maximum width of the ASB are graphically presented. The width of the deformation zone significantly decreases from approximately 95 μm at 0.12 m/s to zero at 17 m/s blanking speed. For the speeds of 0.7 and 8 m/s, the average deformation zone width adjacent to the ASB is approximately 40 μm. The ASB width reaches its maximum at 8 m/s. For the highest blanking speed, the maximum width of the ASB is significantly lower.

Based on the measured values of deformation zone and ASB width, the local shear rates were estimated for the different blanking speeds. For the calculation of the shear zone width the deformation zone width was doubled and added to the ASB width. It has to be pointed out that this method and microstructurally assisted estimation enables only a first approximation of the actual local shear rate, as the determination of the width is limited to the observed microstructural plane in a metallographically polished cross section. Regarding the width of the full ASB, for the blanking conditions at 0.12 and 0.7 m/s as well as at 8 m/s, where failure occurs near or next to the band, the maximum width of the full ASB could be measured. For the condition blanked at 17 m/s, for which failure presumably occurs in the band, the measured width was doubled to estimate the value for the full ASB width. But to be absolutely precise about the shear zone containing ASB and two deformation zones on each ASB side, these would have to be measured on the slug and the blanked sheet at the same failure point. The microstructurally assisted estimated shear zone width and strain rates are listed in Table 2 in comparison to the nominal engineering strain rates. For the blanking speeds of 0.7 and 8 m/s the shear strain rates are in the same range for both calculation methods and the microstructurally assisted estimated shear zone is in good agreement with the clearance. In contrast, for the lowest and the highest blanking speeds these values differ significantly. At 0.12 m/s the microstructurally assisted estimated shear strain rate is halved, when compared to the engineering calculation. Further, the width of the shear zone, determined via measurement on the cross section, is twice the clearance of 100 μm. For the blanking speed of 17 m/s the determined shear zone width is only one fifth of the clearance and the microstructurally assisted estimated shear strain rate is six times higher. This extremely high shear strain rate at 17 m/s promotes thermal softening further, supporting localization of deformation further as well leading to a self-reinforcing system.

**Table 2:** Investigated blanking speeds and resulting average shear rates, calculated for the used clearance of 5% (100 μm).

Blanking speed v in m/s	0.12	0.7	8	17
Engineering shear strain rate γ̇ in s^−1^	1,200	7,000	80,000	170,000
microstructurally assisted estimated shear strain rate γ̇ in s^−1^	609	7861	84,522	1,050,680
microstructurally assisted estimated shear zone width in µm	197	89	95	16

**Fig. 4:**
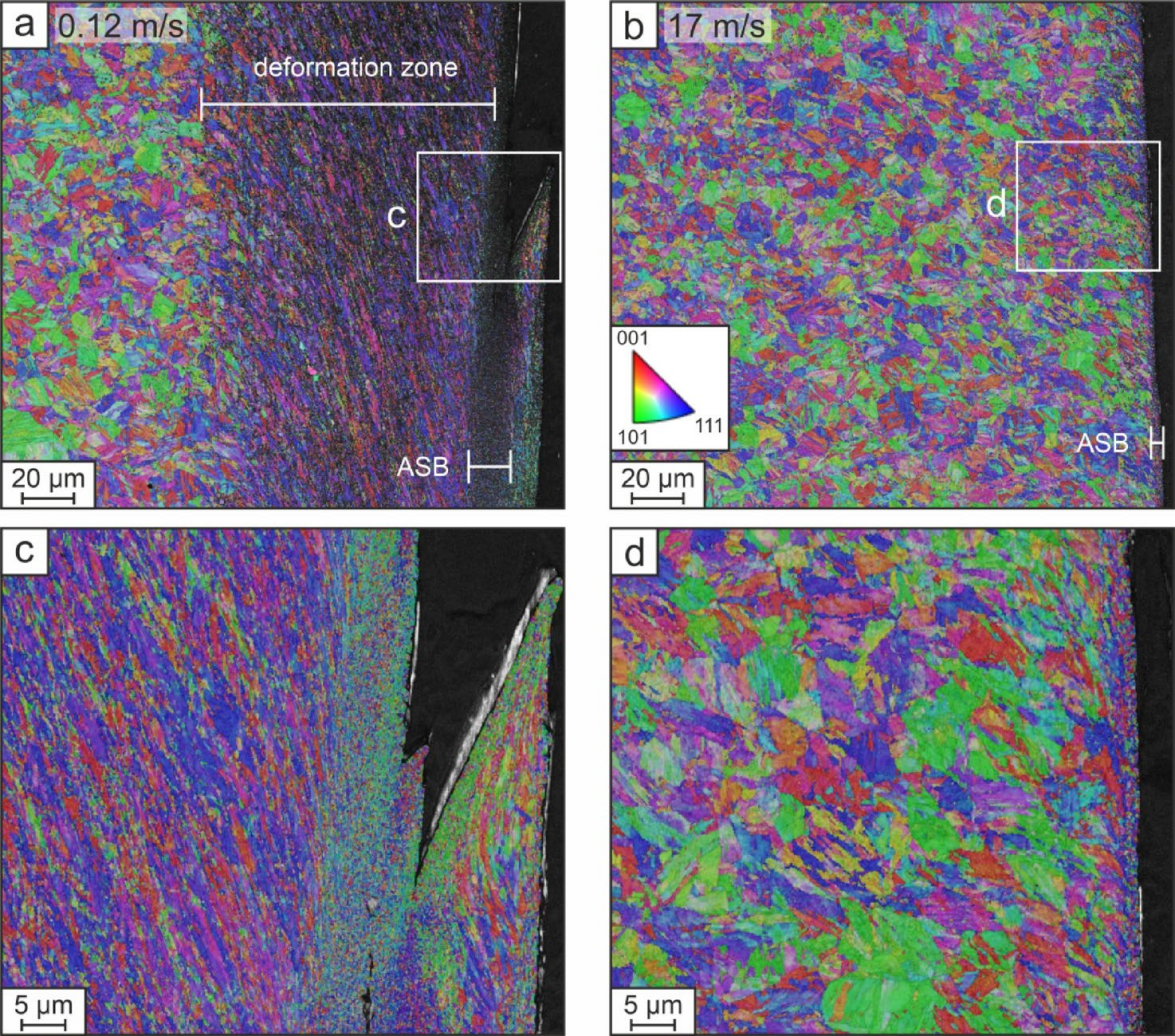
Inverse pole figure maps (IPF-Y) overlayed on band contrast maps for samples blanked at (**a**) 0.12 and (**b**) 17 m/s speed. Detail scans for blanking speeds of (**c**) 0.12 and (**d**) 17 m/s. An increase in blanking speed results in pronounced localization of plastic deformation, resulting in a thin ASB with nanocrystalline grains and no observable deformation zone.

The microstructure of the blanked edges is displayed in the EBSD orientation maps in Fig. 4 for the lowest and highest investigated blanking speeds. For the lowest blanking speed of 0.12 m/s the overview map shows that the induced shear deformation leads to a pronounced deformation zone with high dislocation density (see Fig. 4a). In the detail map (Fig. 4c) the finely recrystallized grains in the ASB are clearly distinguishable from the deformed, and preferentially oriented elongated grains in the deformation zone. In contrast, for the highest blanking speed of 17 m/s the deformation is localized to a narrow region, which results in a thin ASB with recrystallized grains at the blanked edge. For 17 m/s via EBSD no deformation zone is detectable (see Fig. 4b, d).

**Fig. 5:**
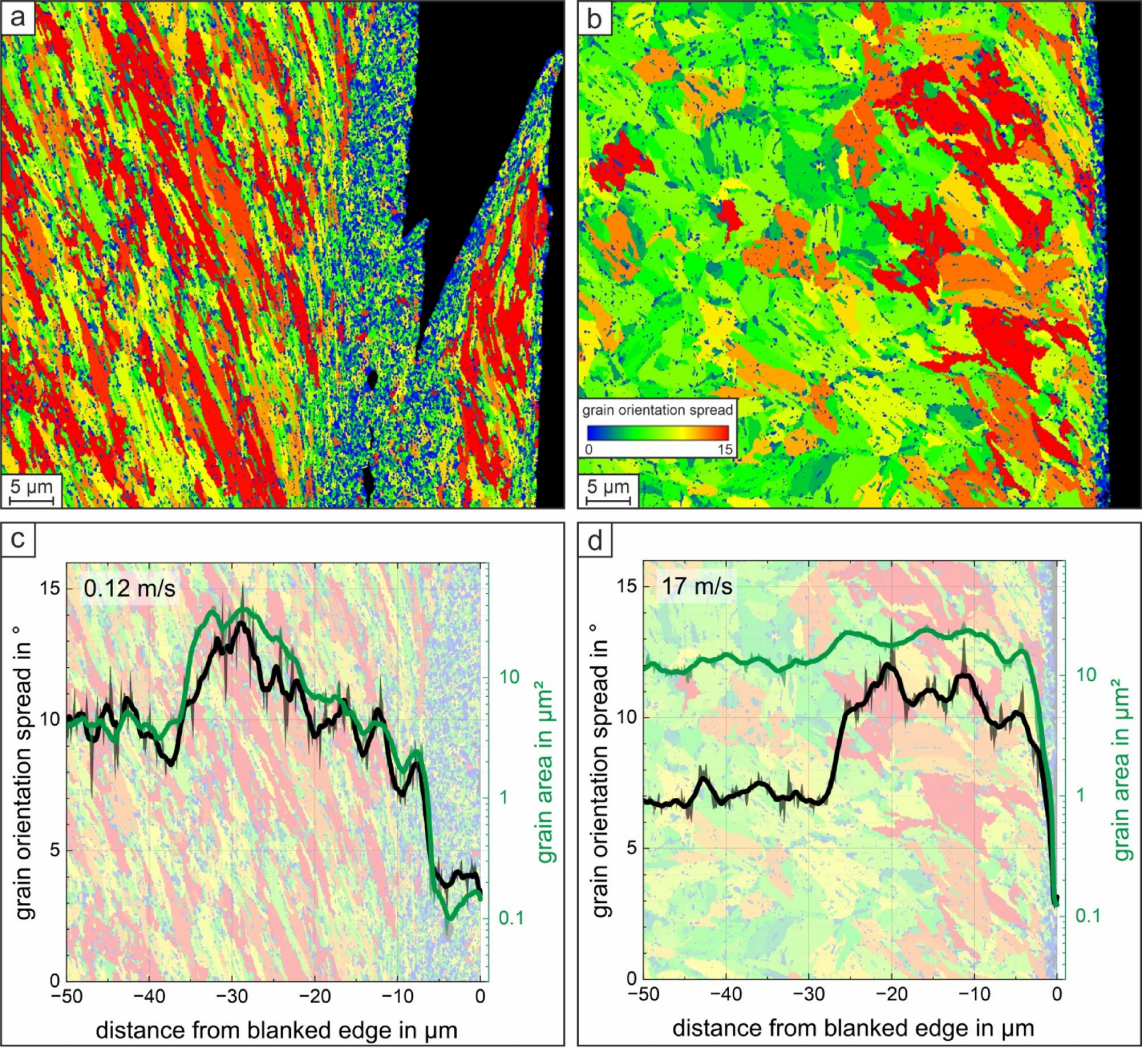
Grain orientation spread (GOS) maps for samples blanked at (**a**) 0.12 and (**b**) 17 m/s speed. Mean GOS and grain area (GA) depending on the distance from blanked edge for (**c**) 0.12 m/s and (**d**) 17 m/s blanking speed (respective measured area from the maps (**a**, **b**) is shown as background). The low GOS combined with the small GA indicate DRX in the ASB for both surfaces, blanked at 0.12 and 17 m/s.

To better visualize the microstructural changes at the blanked edge depending on the blanking speed, for the EBSD detail scans the grain orientation spread (GOS) maps are shown in Fig. 5a, b. In addition, mean values of GOS and grain area (GA) were determined from a series of line measurements. GOS and GA mean values over the distance from the blanked edge are displayed in Fig. 5c, d, with the area used for the line measurements shown as diagram background. For the lowest blanking speed of 0.12 m/s, in the first approximately 7 μm, which corresponds to half the maximum shear band width of this blanked condition (see Fig. 3b), GOS with 4° and GA with 0.1–0.2 μm² are very low. With an increasing distance from the blanked edge both are pronouncedly increased, reaching a maximum at approximately 30 μm distance from the blanked edge with up to 14° GOS and a GA of 12 μm².

For the specimen blanked at 17 m/s GOS and GA exhibiting the same minimum values, when compared to a blanking speed of 0.12 m/s, but the distance from the blanked edge at which minimum values are present are significantly smaller with only approximately 2–3 μm. After this short distance, GOS and GA rapidly increase and reach a plateau value starting at approximately 4 μm distance from the blanked edge. The GA remains almost constant at this plateau value of 11 μm² indicating that the grain size is not significantly affected by this high blanking speed. However, within a distance of 25 μm from the blanked edge the GOS is much higher with 11–12°, when compared to an increasing distance at which the grain orientation decreases to 7°. Further, as due to geometrical constraints the GOS is smaller for smaller grains, the low GOS in combination with the small GA indicate DRX in the ASB for both blanking speeds of 0.12 and 17 m/s, respectively.

**Fig. 6:**
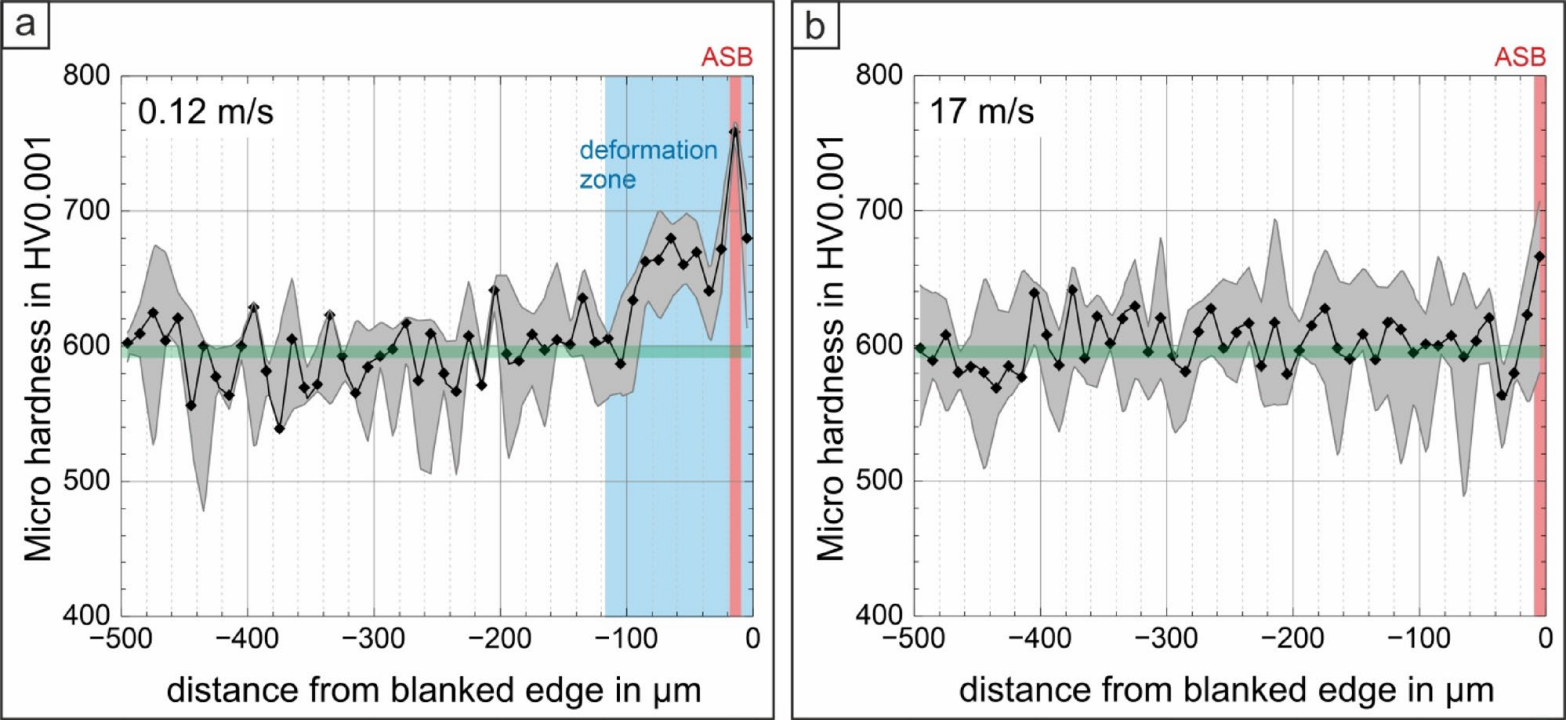
Micro-hardness profiles for the conditions blanked with (**a**) 0.12 m/s and (**b**) 17 m/s, measured on the cross section from the blanked edge (distance 0 μm) towards the undeformed base material (Hardness of base material is marked with green line, deformation zone width in blue and ASB in red). At the lower blanking speed, the hardness increases pronouncedly in the deformation zone. Peak-hardness is achieved in the ASB, which is significantly higher when compared to higher blanking speed.

On the cross sections of these two blanked conditions the micro-hardness depending on the distance from the blanked edge and therefore depending on the local microstructure was determined (see Fig. 6). The median hardness profiles are displayed with minimum and maximum hardness values. As expected, the hardness of the base material is equal for both blanked conditions, as the same sheets were used. The deviation in hardness for the base steel material is explainable by the orientation of the lath-shaped, martensitic microstructure under the indenter. For the blanking speed of 0.12 m/s a pronounced increase in hardness is detectable in the deformation zone. Peak-hardness is reached in the ASB and in the adjacent deformation zone near to blanked edge, where failure occurred, the hardness decreases again. For the condition blanked with 17 m/s the highest hardness is as well determinable in the ASB, but the median hardness in the band of 670 HV0.001 for this condition is significantly lower when compared to peak-hardness in the band of 760 HV0.001 for a blanking speed of 0.12 m/s.

The development of ASB length and ASB and deformation zone width as well as the failure location in correlation with the microstructural features and the hardness of the blanking edge strongly indicates a mechanism change depending on the blanking speed and shear strain rate, respectively. For the blanking speed of 0.12 m/s the deformation induced hardening in this region, observable by the increased micro-hardness and grain refinement in the ASB, presumably overcompensates the lower proportion of thermal softening in the ASB. As a result, the hardness in the band is higher, when compared to the softer surrounding microstructure. Therefore, failure is located near to the ASB in the deformation zone, leading to the jagged appearance of the ASB. With an increase in blanking speed, thermal softening starts to become more relevant and failure occurs more near to the band. At the highest investigated blanking speed, thermal softening in the band seems to be the dominant mechanism for failure. This hypothesis is supported by the hardness of the ASB, which is only slightly higher than the base material due to grain refinement, but much lower when compared to the blanking speed at 0.12 m/s, which leads to the very smooth ASB appearance and overall low roughness of the blanked edge. Further, due to high blanking speed and the high strain rate, respectively, the ASB width is minimal for the investigated conditions. Further, the absence of a deformation zone in the EBSD micrographs indicates that the blanking energy could be used entirely for the transformation of the microstructure by DRX, which is a strong indication for an adiabatic blanking process. The full-length ASB without any deformation zone, formed at a blanking speed of 17 m/s, on a surface produced by an industrial blanking process has to the best of our knowledge not yet been reported by other researchers, and it promises high potential to produce functional surfaces with excellent surface quality and properties by high-speed blanking.

## Conclusions

For a press-hardened 22MnB5 steel sheet, a blanking process was performed using blanking speeds ranging from 0.12 to 17 m/s, resulting in nominal shear rates of up to nearly 100,000 s⁻¹. It is shown that the blanking speed significantly influences the formation of ASBs, which act as precursors to fracture and ultimately develop into the fracture surface. For the highest blanking speed of 17 m/s, an ASB covering the full sheet thickness could be formed. The main findings can be summarized as follows:

1. For low blanking speeds up to 0.7 m/s, short and jagged ASBs and a pronounced deformation zone were formed. As hardening is the dominant mechanism, failure occurs near to ASB and the deformation zone adjacent to the ASB, which results in a high surface roughness, in particular at a blanking speed of 0.12 m/s.
2. Beginning from 8 m/s, with an increase in blanking speed, ASB formation dominates the blanked surface and the geometry of the blanked surface exhibits a pronounced S-shaped profile.
3. In general, an increase in blanking speed leads to an increase in length of the formed ASB on the blanked surface. For a velocity of 17 m/s, for which shear strain rates of 1,050,680 s⁻¹ were determined based on microstructural analysis, the length of the ASB corresponds to the complete sheet thickness. Further, thermal softening appears to be the main mechanism leading to failure and to very smooth ASBs.
4. Blanked surfaces produced by high velocities, where an ASB dominates the blanked surface, exhibit a significantly lower roughness and therefore a higher surface quality, when compared to conventionally blanked surfaces. This may open up high potential to produce functional surfaces by high-speed blanking processes in the future.

## Data Availability

The authors confirm that the data supporting the findings of this study are available within the article.
